# Amino acid polymorphisms in human histocompatibility leukocyte antigen class II and proinsulin epitope have impacts on type 1 diabetes mellitus induced by immune-checkpoint inhibitors

**DOI:** 10.3389/fimmu.2023.1165004

**Published:** 2023-04-06

**Authors:** Hidefumi Inaba, Shuhei Morita, Daisuke Kosugi, Yuki Asai, Yosuke Kaido, Saya Ito, Tomonao Hirobata, Gen Inoue, Yuki Yamamoto, Masatoshi Jinnin, Hiroaki Kimura, Masao Ota, Yuko Okudaira, Hiroyasu Nakatani, Tomoko Kobayashi, Shintaro Iwama, Hiroshi Arima, Takaaki Matsuoka

**Affiliations:** ^1^ Department of Diabetes and Endocrinology, Japanese Red Cross Wakayama Medical Center, Wakayama, Japan; ^2^ The First Department of Medicine, Wakayama Medical University, Wakayama, Japan; ^3^ Department of Dermatology, Wakayama Medical University, Wakayama, Japan; ^4^ Department of Pharmaceutical Health Sciences, Kyushu University of Health and Welfare, Nobeoka, Miyazaki, Japan; ^5^ Department of Medicine, Division of Gastroenterology and Hepatology, Shinshu University School of Medicine, Matsumoto, Japan; ^6^ HLA Typing Section, GenoDive Pharma Inc., Kanagawa, Japan; ^7^ Research Department, Biospecimen Laboratories, Inc. Tokyo, Japan; ^8^ Department of Endocrinology and Diabetes, Nagoya University Graduate School of Medicine, Nagoya, Aichi, Japan

**Keywords:** human histocompatibility leukocyte antigen, immune-checkpoint inhibitors, immune-related adverse events, type 1 diabetes mellitus, proinsulin

## Abstract

**Introduction:**

Immune-checkpoint inhibitors are effective in various advanced cancers. Type 1 diabetes mellitus induced by them (ICI-T1DM) is a serious complication requiring prompt insulin treatment, but the immunological mechanism behind it is unclear.

**Methods:**

We examined amino acid polymorphisms in human histocompatibility leukocyte antigen (HLA) molecules and investigated proinsulin epitope binding affinities to HLA molecules.

**Results and Discussion:**

Twelve patients with ICI-T1DM and 35 patients in a control group without ICI-T1DM were enrolled in the study. Allele and haplotype frequencies of HLA-DRB1*04:05, DQB1*04:01, and most importantly DPB1*05:01 were significantly increased in patients with ICI-T1DM. In addition, novel amino acid polymorphisms in HLA-DR (4 polymorphisms), in DQ (12 polymorphisms), and in DP molecules (9 polymorphisms) were identified. These amino acid polymorphisms might be associated with the development of ICI-T1DM. Moreover, novel human proinsulin epitope clusters in insulin A and B chains were discovered *in silico* and *in vitro* peptide binding assays to HLA-DP5. In conclusion, significant amino acid polymorphisms in HLA-class II molecules, and conformational alterations in the peptide-binding groove of the HLA-DP molecules were considered likely to influence the immunogenicity of proinsulin epitopes in ICI-T1DM. These amino acid polymorphisms and HLA-DP5 may be predictive genetic factors for ICI-T1DM.

## Introduction

Immune-checkpoint inhibitors (ICIs) are effective agents in various cancers; however, immune-related adverse events (irAEs) often occur during treatment with ICIs (1–5). Major endocrine irAEs include pituitary irAE (3), thyroid irAE (4), and ICI-induced type 1 diabetes mellitus (ICI-T1DM). ICI-T1DM is an especially critical irAE due to the possibility of acute damage to pancreatic β-cells (5). Qiu et al. reported that anti-insulin antibody was observed in 7 patients with ICI-T1DM (3 patients with fulminant type 1 diabetes, and 4 patients with acute type 1 diabetes) (6). One patient with ICI-T1DM exhibited anti-insulin antibody positivity in our study (5). Proinsulin is cleaved into insulin and C-peptide in pancreatic β-cells at secretion (7), we thus hypothesized that proinsulin could be a major autoantigen in ICI-T1DM.

We have previously identified thyrotropin receptor epitopes to HLA-DR molecules in Graves’ disease *in silico*, *in vitro*, and human studies (8, 9). Further, we have also previously in part examined of histocompatibility leukocyte antigen (HLA) alleles and haplotypes in ICI-T1DM. However, immunological mechanisms in ICI-T1DM are largely unknown due to the rarity of the disease (0.8% prevalence among ICI-treated patients (5).

In the current study, novel amino acid polymorphisms in HLA class II molecules in patients with ICI-T1DM, and *in vitro* proinsulin epitope binding affinities to HLA-DP molecules were revealed. These genetic factors may be utilized for prediction of ICI-T1DM, and also contribute to elucidate the mechanism of cancer immunotherapy and ICI-T1DM. Therefore, current study offers novel management and monitoring options for cancer immunotherapy.

## Patients and methods

### Patients

Patients were recruited from the Japanese Red Cross Society Wakayama Medical Center (JRCW), Wakayama Medical University Hospital (WMU), and Nagoya University (NU). Patients with advanced malignant diseases who received ICI treatment were examined during 2016-2021. ICI treatments included anti-PD-1 antibody (nivolumab or pembrolizumab), anti-PD-L1 antibody (durvalumab or atezolizumab), or anti-CTLA-4 antibody (ipilimumab) following nivolumab (5, 10). The study protocol was approved by the JRCW, WMU, and NU Institutional Ethical Review Boards, and written informed consent was obtained from all participants.

### Assessment of irAEs and ICI-T1DM

Assessment of irAEs was made based on the descriptions and grading scales of the *National Cancer Institute (NCI) Common Terminology Criteria for Adverse Events version 3.0*. Diagnostic criteria for T1DM were based on the hyperglycemic symptoms, and continuous requirement of insulin therapy irrespective of autoimmune diabetes-related autoantibodies (11). ICI-controls were defined as those who were treated with ICI but did not develop any irAEs including T1DM (5, 10). Healthy Japanese individuals were used as general controls (12–14), and participants had no clinical or demographic differences and they had the same ethnic background.

### HLA-genotyping and amino acid sequences

DNA extracted from blood was genotyped in HLA-*A, B, C, DRB1, DQB1*, and *DPB1* alleles by the next-generation sequence method and a Luminex system with WAKFlow HLA typing kits (GenoDive Pharma, Kanagawa, Japan) as previously described (5, 12–14). Allele frequencies were determined by direct counting and three-locus (DRB1~DQB1~DPB1) haplotype frequencies were obtained by maximum likelihood methods as previously described (http://cmpg.unibe.ch/software/arlequin3/) (5, 12–14). Amino acid sequences were downloaded from (https://www.ebi.ac.uk/ipd/imgt/hla/).

### Prediction of human proinsulin-peptides binding affinities to HLA-DP5 and HLA-DP15

*In silico* binding of human proinsulin (AA 1-110) (NP_000198.1) derived peptide to HLA-DP5 molecule (HLA-DPA1*02:02, HLA-DPB1*05:01) and a control allele, HLA-DP15 molecule (HLA-DPA1*02:02, HLA-DPB1*15:01: not previously reported as susceptible or protective allele) were predicted by NetMHCIIpan software version 4.0. The %RANK threshold for strong binders was set as <5%. The peptides with a 5-50%RANK threshold were set as intermediate binders.

### *In vitro* human proinsulin peptides binding assay to HLA-DP5 and HLA-DP15

Fifteen-mer peptides derived from human proinsulin were synthesized based on the predicted affinities to HLA-DP5: 1) strong binders, 2) intermediate binders, and 3) others to cover the entire sequence (PEPscreen^®^ peptide library, ProImmune, Oxford, UK). Peptides that were known to have high affinities with HLA-DP5 and HLA-DP15 were also synthesized. The synthesized peptides were subjected to an *in vitro* peptide binding assay (ProImmune REVEAL^®^ MHC class II-peptide binding assay: (ProImmune, Oxford, UK) using recombinant HLA-DP5 and HLA-DP15 proteins.Detection of binding peptides is based on the presence or absence of the native conformation of the MHC-peptide complex in an immunoassay. Each test peptide was given a ‘REVEAL SCORE’ relative to positive control peptides, which were known to bind HLA-DP5 or HLA-DP15 with high affinity (signal of known positive control peptide which was known to bind each HLA-DP with high affinity, divided by each test peptide x 100%). The high-throughput assay quantifies the ability of the test peptides to bind to HLA-DP5 and HLA-DP15. The results of the three separate assays were in close agreement and are presented as an average of the results.

### Three-dimensional modeling of the HLA-class II molecules

Three-dimensional modeling of HLA-class II molecules (HLA-DR, HLA-DQ, and HLA-DP) was downloaded from the Protein Data Bank database (15), and visualized with PyMOL (16).

### Statistical analysis

Differences between the two groups were analyzed by a Mann-Whitney U test. The association of allele frequencies was analyzed using Fisher’s exact test. Frequencies of HLA alleles and amino acid polymorphisms were analyzed by univariate and multivariate logistic regression analysis with stepwise selection of covariates. Bonferroni test was applied if the variables were significant. Statistical analyses were performed using JMP, version 15 (SAS Institute Inc., Cary, N.C., USA). P values < 0.05 were considered to be statistically significant.

## Results

### Clinical characteristics of patients with ICI-T1DM

A total of twelve patients with ICI-T1DM (six patients from JRC, two patients from WMU, and four patients from NU) were identified and enrolled in the study (**Tables 1A**, **1B**; **Supplementary Table 1**). Clinical profiles of seven patients with ICI-T1DM were partly described previously (5). Thirty-five independent ICI-controls (13 from WMU and 22 from NU) were prospectively identified as subjects without irAE and were analyzed. Anti-GAD65 antibody was negative for patients measured. Patient #6 only developed insulin autoantibodies (IAA).

**Table 1A:** Clinical characteristics of the patients with ICI-T1DM.

Patient	Age (year)	Gender	Cancer	ICI	Number of ICI treatment cycle	Time of onset of ICI-T1DM (weeks)	HbA1c at onset (%)	Casual PG at onset (mg/dl)	PH of diabetes	Other irAEs	Comorbidities	Anti-GAD65 Ab/Insulin Ab	Tumor response	Continuation of ICI
1	70	M	NSCLC	P	3	9	6	**564**	No	Eczema	Dyslipidemia	Ne/Ne	PR	Continued
2	80	M	NSCLC	P	11	37	**7.3**	**420**	No	IP, Grade1	Hypertension	Ne/Ne	PR	Continued
3	79	M	NSCLC	P	4	12	5.3	**404**	No	None	Hashimoto's thyroiditis	Ne/ND	PR	Discontinued
4	71	M	NSCLC	P	9	31	**8.7**	**491**	T2DM	None	Hypertension	Ne/Ne	PR	Continued
5	72	M	SCLC	D	2	6	**6.5**	**502**	No	None	None	Ne/Ne	PR	Continued
6	80	F	MM	N/Ipi	N, 20 cycles, then Ipi once	N 60w, Ipi 3w, total 63	**7.7**	**639**	No	None	Hypertension	Ne/Po	CR	Continued
7	78	M	MM	N	14	29	**8.5**	**940**	No	None	Hypertension	Ne/Ne	CR	Continued
8	70	F	RCC	P	4	40	**10.6**	**616**	T2DM	THY	Hypertension	Ne/ND	PR	Discontinued
9	75	M	NSCLC	P	7	29	7.6	**684**	T2DM	None	Atrial fibrillation	Ne/Ne	PD	Discontinued
10	71	F	MM	N	9	21	6.3	**489**	No	None	Hypertension	Ne/Ne	PD	Continued
11	66	M	NSCLC	N/A	N, 18 cycles, then A once	N 75w, A 2w, total 77w	6.9	**1041**	No	None	Hyperuricemia	Ne/Ne	PD	Continued
12	55	M	NSCLC	N	51	121	9.4	**278**	T2DM	None	None	Ne/Ne	PD	Discontinued

M, male; F, female; Cancer, underlying cancer; ICI, type of ICI; P, pembrolizumab; N, nivolumab; Ipi, ipilimumab; D, durvalumab; A, atezolizumab; N/Ipi, N then ipi; N/A, N then A.

PH, past history; PG, plasma glucose; T2DM, type 2 diabetes mellitus.

MM, malignant melanoma; NSCLC, Non-small cell lung cancer; SCLC, small cell lung cancer; RCC, renal cell carcinoma; IP, interstitial pneumonitis; THY, thyroiditis.

ND, not determined; Ab, autoantibody; Ne, Negative; Po, Positive; CR, complete response; PR, partial response; PD, progressive disease.

Abnormal values are shown in bold. Partial data of patients 1-7 was previously reported in ref (5).

**Table 1B:** The plasma glucose levels of ICI-T1DM patients and their summarized HLA typing results.

Patient	Casual PG at onset (mg/dl)	HLA-DRB1		HLA-DQB1		HLA-DPB1		HLA-DR-DQ haplotype 1		HLA-DR-DQ haplotype 2		HLA-DR-DQ-DP haplotype 1			HLA-DR-DQ-DP haplotype 2		
1	**564**	*04:05	*09:01	*03:03	*04:01	*05:01	*05:01	DRB1*04:05	DQB1*04:01	DRB1*09:01	DQB1*03:03	DRB1*04:05	DQB1*04:01	DPB1*05:01	DRB1*09:01	DQB1*03:03	DPB1*05:01
2	**420**	*04:05	*04:06	*03:02	*04:01	*02:01	*05:01	DRB1*04:05	DQB1*04:01	DRB1*04:06	DQB1*03:02	DRB1*04:05	DQB1*04:01	DPB1*05:01	DRB1*04:06	DQB1*03:02	DPB1*02:01
3	**404**	*09:01	*14:54	*03:03	*05:03	*04:02	*05:01	DRB1*09:01	DQB1*03:03	DRB1*14:54	DQB1*05:03	DRB1*09:01	DQB1*03:03	DPB1*04:02	DRB1*14:54	DQB1*05:03	DPB1*05:01
4	**491**	*04:05	*08:02	*03:02	*04:01	*05:01	*19:01	DRB1*04:05	DQB1*04:01	DRB1*08:02	DQB1*03:02	DRB1*04:05	DQB1*04:01	DPB1*19:01	DRB1*08:02	DQB1*03:02	DPB1*05:01
5	**502**	*04:05	*08:03	*04:01	*06:01	*05:01	*05:01	DRB1*04:05	DQB1*04:01	DRB1*08:03	DQB1*06:01	DRB1*04:05	DQB1*04:01	DPB1*05:01	DRB1*08:03	DQB1*06:01	DPB1*05:01
6	**639**	*04:05	*12:01	*03:01	*04:01	*05:01	*05:01	DRB1*04:05	DQB1*04:01	DRB1*12:01	DQB1*03:01	DRB1*04:05	DQB1*04:01	DPB1*05:01	DRB1*12:01	DQB1*03:01	DPB1*05:01
7	**940**	*08:03	*08:03	*06:01	*06:01	*05:01	*05:01	DRB1*08:03	DQB1*06:01	DRB1*08:03	DQB1*06:01	DRB1*08:03	DQB1*06:01	DPB1*05:01	DRB1*08:03	DQB1*06:01	DPB1*05:01
8	**616**	*04:05	*11:01	*03:01	*04:01	*02:01	*05:01	DRB1*04:05	DQB1*04:01	DRB1*11:01	DQB1*03:01	DRB1*04:05	DQB1*04:01	DPB1*05:01	DRB1*11:01	DQB1*03:01	DPB1*02:01
9	**684**	*09:01	*14:03	*03:01	*03:03	*05:01	*05:01	DRB1*09:01	DQB1*03:03	DRB1*14:03	DQB1*03:01	DRB1*09:01	DQB1*03:03	DPB1*05:01	DRB1*14:03	DQB1*03:01	DPB1*05:01
10	**489**	*09:01	*15:02	*03:03	*06:01	*02:01	*09:01	DRB1*09:01	DQB1*03:03	DRB1*15:02	DQB1*06:01	DRB1*09:01	DQB1*03:03	DPB1*02:01	DRB1*15:02	DQB1*06:01	DPB1*09:01
11	**1041**	*04:05	*13:02	*04:01	*06:04	*05:01	*05:01	DRB1*04:05	DQB1*04:01	DRB1*13:02	DQB1*06:04	DRB1*04:05	DQB1*04:01	DPB1*05:01	DRB1*13:02	DQB1*06:04	DPB1*05:01
12	**278**	*04:05	*11:01	*03:01	*04:01	*05:01	*14:01	DRB1*04:05	DQB1*04:01	DRB1*11:01	DQB1*03:01	DRB1*04:05	DQB1*04:01	DPB1*05:01	DRB1*11:01	DQB1*03:01	DPB1*14:01

PG, plasma glucose; Abnormal values and HLA-DRB1*04:05, DQB1*04:01, and DPB1*05:01 are shown in bold.

### HLA alleles and haplotypes analysis

The plasma glucose levels of patients with ICI-T1DM and HLA typing results are summarized in **Table 1B**. We have used HLA-class I and II four-digit allelic typing results in the allele or haplotype analysis (**Tables 2A**–**E**; **Supplementary Table 2A–C**). Allele frequencies of HLA-DRB1*04:05 (**Table 2A**) and HLA-DQB1*04:01 (**Table 2B**), both alleles in complete linkage disequilibrium, were significantly higher in patients with ICI-T1DM than in general controls and also in ICI-controls. HLA-DPB1*05:01 allele frequency was more significantly associated with an increased risk of ICI-T1DM when compared with general controls and also in ICI-controls (P=0.005 and 0.004, respectively) (**Table 2C**).

**Table 2A:** Allele frequencies of HLA-DR in patients with ICI-T1DM and controls.

	ICI-T1DM (*N*=24)		ICI-Controls (*N*=70)		^*1^Controls (*N*=618644)	ICI-T1DM vs general controls				ICI-T1DM vs ICI-controls				ICI-controls vs general controls		
allele	*n*	F (%)	*n*	F (%)	F (%)	^*2^ P	^*3^ Pc	OR	95% CI	^*2^ P	^*3^ Pc	OR	95% CI	^*2^ P	OR	95% CI
DRB1*01:01	0	0.0	5	7.1	5.65	NS				NS				NS		
DRB1*04:01	0	0.0	0	0.0	1.03	NS				NS				NS		
DRB1*04:03	0	0.0	5	7.1	3.13	NS				NS				NS		
DRB1*04:05	8	33.3	5	7.1	13.41	**0.03**	NS	3.34	1.26--9.20	**0.003**	NS	6.50	1.95--21.60	NS		
DRB1*04:06	1	4.2	3	4.3	3.28	NS				NS				NS		
DRB1*04:10	0	0.0	2	2.9	2.12	NS				NS				NS		
DRB1*08:02	1	4.2	4	5.7	4.29	NS				NS				NS		
DRB1*08:03	3	12.5	5	7.1	7.93	NS				NS				NS		
DRB1*09:01	4	16.7	8	11.4	14.6	NS				NS				NS		
DRB1*10:01	0	0.0	1	1.4	0.48	NS				NS				NS		
DRB1*11:01	2	8.3	2	2.9	2.49	NS				NS				NS		
DRB1*11:06	0	0.0	1	1.4	0.002	NS				NS				NS		
DRB1*12:01	1	4.2	3	4.3	3.68	NS				NS				NS		
DRB1*12:02	0	0.0	2	2.9	1.69	NS				NS				NS		
DRB1*13:02	1	4.2	6	8.6	6.34	NS				NS				NS		
DRB1*14:03	1	4.2	1	1.4	1.63	NS				NS				NS		
DRB1*14:05	0	0.0	2	2.9	2.14	NS				NS				NS		
DRB1*14:06	0	0.0	0	0.0	1.54	NS				NS				NS		
DRB1*14:54	1	4.2	0	0.0	3.49	NS				NS				NS		
DRB1*15:01	0	0.0	8	11.4	7.88	NS				NS				NS		
DRB1*15:02	1	4.2	6	8.6	10.27	NS				NS				NS		
DRB1*16:02	0	0.0	1	1.4	0.82	NS				NS				NS		
Others	0	0.0	0	0.0	2.11	NS				NS				NS		
total	24	100.00	70	100.00	100.00											

Alleles with frequencies more than 1.0% in controls were included to the analysis (22 alleles). N, n, number of the alleles.

F, frequency of the allele; OR, odds ratio; CI, confidence interval; NS, not significant.

P values less than 0.05 are shown in bold.

*1 General control subjects: Japanese Society for Histocompatibility and Immunogenetics: http://jshi.umin.ac.jp/standarization/file/JSHI-hyokiallele-2022list.pdf :JSHI2022 ref (12).

http://jshi.umin.ac.jp/standarization/file/JSHI-hyokiallele-2022list.pdf

*2 Each allele frequency was analyzed using Fisher’s exact test with 2 x2 contingency tables.

*3 Pc: Bonferroni correction.

95%CI: 95% confidence intervals.

**Table 2B:** Allele frequencies of HLA-DQB1 in patients with ICI-T1DM and controls.

	ICI-T1DM (*N*=24)		ICI-Controls (*N*=70)		^*1^Controls (*N*=1483)	ICI-T1DM vs general controls				ICI-T1DM vs ICI-controls				ICI-controls vs general controls		
allele	*n*	F (%)	*n*	F (%)	F (%)	^*2^ P	^*3^ Pc	OR	95% CI	^*2^ P	^*3^ Pc	OR	95% CI	^*2^ P	OR	95% CI
DQB1*03:01	4	16.7	6	8.6	11.43	NS				NS				NS		
DQB1*03:02	2	8.3	10	14.3	9.59	NS				NS				NS		
DQB1*03:03	4	16.7	11	15.7	15.54	NS				NS				NS		
DQB1*04:01	8	33.3	5	7.1	12.9	**0.03**	NS	3.34	1.26--9.20	**0.003**	**0.033**	6.50	1.95--21.60	NS		
DQB1*04:02	0	0.0	4	5.7	4.21	NS				NS				NS		
DQB1*05:01	0	0.0	5	7.1	6.58	NS				NS				NS		
DQB1*05:02	0	0.0	1	1.4	2.64	NS				NS				NS		
DQB1*05:03	1	4.2	3	4.3	3.94	NS				NS				NS		
DQB1*06:01	4	16.7	11	15.7	19.08	NS				NS				NS		
DQB1*06:02	0	0.0	8	11.4	7.15	NS				NS				NS		
DQB1*06:04	1	4.2	6	8.6	5.18	NS				NS				NS		
Others	0	0	0	0.0	1.76	NS				NS				NS		
total	24	100.00	70	100.00	100.00											

Alleles with frequencies more than 1.0% in controls were included to the analysis (11 alleles). N, n, number of the alleles.

F, frequency of the allele; OR, odds ratio; CI, confidence interval; NS, not significant.

P values less than 0.05 are shown in bold.

*1 General control subjects: Japanese Society for Histocompatibility and Immunogenetics: http://jshi.umin.ac.jp/standarization/file/JSHI-hyokiallele-2022list.pdf :JSHI2022 ref (12).

http://jshi.umin.ac.jp/standarization/file/JSHI-hyokiallele-2022list.pdf

*2 Each allele frequency was analyzed using Fisher’s exact test with 2 x2 contingency tables.

*3 Pc: Bonferroni correction.

95%CI: 95% confidence intervals.

**Table 2C:** Allele frequencies of HLA-DPB1 in patients with ICI-T1DM and controls.

	ICI-T1DM (*N*=24)		ICI-Controls (*N*=70)		^*1^Controls (*N*=1483)	ICI-T1DM vs general controls				ICI-T1DM vs ICI-controls				ICI-controls vs general controls		
allele	*n*	F (%)	*n*	F (%)	F (%)	^*2^ p	^*3^ Pc	OR	95% CI	^*2^ p	^*3^ Pc	OR	95% CI	^*2^ p	OR	95% CI
DPB1*02:01	3	12.5	17	24.3	24.11	NS				NS				NS		
DPB1*02:02	0	0.0	6	8.6	3.41	NS				NS				NS		
DPB1*03:01	0	0.0	6	8.6	3.98	NS				NS				NS		
DPB1*04:01	0	0.0	6	8.6	5.06	NS				NS				NS		
DPB1*04:02	1	4.2	7	10.0	9.78	NS				NS				NS		
DPB1*05:01	17	70.8	24	34.3	38.4	**0.005**	NS	3.96	1.54--10.18	**0.004**	**0.04**	4.66	1.73--12.48	NS		
DPB1*09:01	1	4.2	2	2.9	9.95	NS				NS				NS		
DPB1*13:01	0	0.0	1	1.4	1.96	NS				NS				NS		
DPB1*14:01	1	4.2	1	1.4	1.48	NS				NS				NS		
DPB1*19:01	1	4.2	0	0.0	0.74	NS				NS				NS		
Others	0	0.0	0	0.0	1.13	NS				NS				NS		
total	24	100.00	70	100.00	100.00											

Alleles with frequencies more than 1.0% in controls were included to the analysis (10 alleles). N, n, number of the alleles.

F, frequency of the allele; OR, odds ratio; CI, confidence interval; NS, not significant.

P values less than 0.05 are shown in bold.

*1 General control subjects: Japanese Society for Histocompatibility and Immunogenetics: http://jshi.umin.ac.jp/standarization/file/JSHI-hyokiallele-2022list.pdf :JSHI2022 ref (12).

http://jshi.umin.ac.jp/standarization/file/JSHI-hyokiallele-2022list.pdf

*2 Each allele frequency was analyzed using Fisher’s exact test with 2 x2 contingency tables.

*3 Pc: Bonferroni correction.

95%CI: 95% confidence intervals.

**Table 2D:** Haplotype frequencies of HLA-DRB1-DQB1 in patients with ICI-T1DM.

Haplotype	ICI-T1DM (*N*=24)		ICI-Controls (*N*=70)		^*1^Controls (*N*=2992)	ICI-T1DM vs general controls				ICI-T1DM vs ICI-controls				ICI-controls vs general controls		
DRB1-DQB1	*n*	F (%)	*n*	F (%)	F (%)	^*2^ P	^*3^ Pc	OR	95% CI	^*2^ P	^*3^ Pc	OR	95% CI	^*2^ P	OR	95% CI
*01:01*05:01	0	0.0	4	5.7	6.05	NS				NS				NS		
*01:01*05:03	0	0.0	1	1.4	0.00	NS				NS				NS		
*04:01-*03:01	0	0.0	0	0.0	1.00	NS				NS				NS		
*04:03-*03:02	0	0.0	5	7.1	2.67	NS				NS				NS		
*04:05-*04:01	8	33.3	5	7.1	12.83	**0.026**	NS	3.67	1.33--10.19	**0.003**	NS	6.20	1.95--21.60	NS		
*04:06-*03:02	1	4.2	3	4.3	3.14	NS				NS				NS		
*04:10-*04:02	0	0.0	2	2.9	1.84	NS				NS				NS		
*08:02-*03:02	1	4.2	2	2.9	2.51	NS				NS				NS		
*08:02-*04:02	0	0.0	2	2.9	2.34	NS				NS				NS		
*08:03-*06:01	3	12.5	5	7.1	8.16	NS				NS				NS		
*09:01-*03:03	4	16.7	8	11.4	14.47	NS				NS				NS		
*10:01-*05:01	0	0.0	1	1.4	0.50	NS				NS				NS		
*11:01-*03:01	2	8.3	1	1.4	2.74	NS				NS				NS		
*11:01-*03:03	0	0.0	1	1.4	0.07	NS				NS				NS		
*11:06-*03:01	0	0.0	1	1.4	0.00	NS				NS				NS		
*12:01-*03:01	1	4.2	4	5.7	2.61	NS				NS				NS		
*12:01-*03:03	0	0.0	1	1.4	0.84	NS				NS				NS		
*12:02-*03:01	0	0.0	0	0.0	1.87	NS				NS				NS		
*13:02-*06:04	1	4.2	6	8.6	5.18	NS				NS				NS		
*14:03-*03:01	1	4.2	1	1.4	1.20	NS				NS				NS		
*14:05-*05:03	0	0.0	2	2.9	1.97	NS				NS				NS		
*14:06-*03:01	0	0.0	0	0.0	1.24	NS				NS				NS		
*14:54-*05:02	0	0.0	0	0.0	1.64	NS				NS				NS		
*14:54-*05:03	1	4.2	0	0.0	1.94	NS				NS				NS		
*15:01-*06:02	0	0.0	9	12.9	7.15	NS				NS				NS		
*15:02-*06:01	1	4.2	5	7.1	10.86	NS				NS				NS		
*16:02-*05:02	0	0.0	1	1.4	0.77	NS				NS				NS		
Others	0	0.0	0	0.0	4.41	NS				NS				NS		
total	24	100.00	70	100.00	100.00											

Haplotypes with frequencies more than 1.0% in controls were included to the analysis (27 haplotypes). N, n, number of the haplotypes.

*1 Control subjets: HLA LABORATORY, Japan INC: http://hla.or.jp/med/frequency_search/ja/haplo/ ref (11).

*2 Each haplotype frequency was analyzed using Fisher’s exact test with 2 x2 contingency tables.

*3 Pc: Bonferroni correction.

F, frequency of the haplotype; NS, not significant.

P values less than 0.05 are shown in bold.

95%CI: 95% confidence intervals.

**Table 2E:** Haplotype frequencies of HLA-DRB1-DQB1-DPB1 in patients with ICI-T1DM.

Haplotype	ICI-T1DM (*N*=24)		ICI-Controls (*N*=70)		^*1^Controls (*N*=2938)	ICI-T1DM vs general controls				ICI-T1DM vs ICI-controls				ICI-controls vs general controls		
DRB1-DQB1-DPB1	*n*	F (%)	*n*	F (%)	F (%)	^*2^ P	^*3^ Pc	OR	95% CI	^*2^ P	^*3^ Pc	OR	95% CI	^*2^ P	OR	95% CI
*15:02-*06:01-*09:01	1	4.2	2	2.86	8.88	NS				NS				NS		
*04:05-*04:01-*05:01	7	29.2	3	4.29	7.32	**0.006**	NS	5.47	1.77--17.04	**0.002**	NS	9.20	2.31--36.13	NS		
*09:01-*03:03-*05:01	2	8.3	4	5.71	6.77	NS				NS				NS		
*09:01-*03:03-*02:01	1	4.2	1	1.43	5.28	NS				NS				NS		
*01:01-*05:01-*04:02	0	0.0	4	5.71	4.08	NS				NS				NS		
*13:02-*06:04-*04:01	0	0.0	5	7.14	3.68	NS				NS				NS		
*08:03-*06:01-*05:01	3	12.5	3	4.29	3.54	NS				NS				NS		
*15:01-*06:02-*02:01	0	0.0	2	2.86	3.06	NS				NS				NS		
*15:01-*06:02-*05:01	0	0.0	3	4.29	3.03	NS				NS				NS		
*04:05-*04:01-*02:01	0	0.0	0	0.00	2.28	NS				NS				NS		
*08:03-*06:01-*02:01	0	0.0	0	0.00	1.91	NS				NS				NS		
*08:02-*03:02-*05:01	1	4.2	2	2.86	1.84	NS				NS				NS		
*08:03-*06:01-*02:02	0	0.0	2	2.86	1.60	NS				NS				NS		
*04:05-*04:01-*04:02	0	0.0	0	0.00	1.53	NS				NS				NS		
*12:01-*03:01-*05:01	1	4.2	0	0.00	1.43	NS				NS				NS		
*04:06-*03:02-*02:01	1	4.2	2	2.86	1.33	NS				NS				NS		
*04:06-*03:02-*05:01	0	0.0	1	1.43	1.12	NS				NS				NS		
*12:02-*03:01-*05:01	0	0.0	1	1.43	1.09	NS				NS				NS		
*11:01-*03:01-*05:01	0	0.0	1	1.43	1.06	NS				NS				NS		
*01:01-*05:01-*05:01	0	0.0	1	1.43	1.02	NS				NS				NS		
*04:03-*03:02-*02:01	0	0.0	1	1.43	1.02	NS				NS				NS		
*11:01-*03:01-*02:01	1	4.2	0	0.00	0.99	NS				NS				NS		
*14:03-*03:01-*05:01	1	4.2	1	1.43	0.95	NS				NS				NS		
*15:02-*06:01-*02:01	0	0.0	3	4.29	0.92	NS				NS				NS		
*14:54-*05:03-*05:01	1	4.2	0	0.00	0.85	NS				NS				NS		
*04:05-*04:01-*03:01	0	0.0	1	1.43	0.75	NS				NS				NS		
*12:01-*03:03-*05:01	0	0.0	1	1.43	0.75	NS				NS				NS		
*15:02-*06:01-*05:01	0	0.0	1	1.43	0.72	NS				NS				NS		
*09:01-*03:03-*04:02	1	4.2	2	2.86	0.65	NS				NS				NS		
*14:05-*05:03-*02:01	0	0.0	2	2.86	0.65	NS				NS				NS		
*08:02-*04:02-*05:01	0	0.0	1	1.43	0.58	NS				NS				NS		
*04:10-*04:02-*03:01	0	0.0	2	2.86	0.55	NS				NS				NS		
*08:02-*04:02-*02:01	0	0.0	1	1.43	0.51	NS				NS				NS		
*15:01-*06:02-*13:01	0	0.0	1	1.43	0.44	NS				NS				NS		
*12:02-*03:01-*02:01	0	0.0	1	1.43	0.37	NS				NS				NS		
*04:05-*04:01-*19:01	1	4.2	0	0.00	0.37	NS				NS				NS		
*13:02-*06:04-*05:01	1	4.2	0	0.00	0.36	NS				NS				NS		
*04:03-*03:02-*03:01	0	0.0	1	1.43	0.34	NS				NS				NS		
*16:02-*05:02-*02:02	0	0.0	1	1.43	0.31	NS				NS				NS		
*12:01-*03:01-*02:01	0	0.0	1	1.43	0.31	NS				NS				NS		
*15:01-*06:02-*03:01	0	0.0	1	1.43	0.27	NS				NS				NS		
*09:01-*03:03-*02:02	0	0.0	1	1.43	0.24	NS				NS				NS		
*10:01-*05:01-*02:01	0	0.0	1	1.43	0.24	NS				NS				NS		
*04:05-*04:01-*02:02	0	0.0	1	1.43	0.17	NS				NS				NS		
*04:03-*03:02-*04:02	0	0.0	1	1.43	0.14	NS				NS				NS		
*04:05-*04:01-*14:01	0	0.0	0	0.00	0.10	NS				NS				NS		
*15:01-*06:02-*04:01	0	0.0	1	1.43	0.07	NS				NS				NS		
*04:03-*03:02-*02:02	0	0.0	1	1.43	0.03	NS				NS				NS		
*12:01-*03:03-*02:01	0	0.0	1	1.43	0.03	NS				NS				NS		
*13:02-*06:04-*03:01	0	0.0	1	1.43	0.03	NS				NS				NS		
*11:01-*03:03-*14:01	1	4.2	1	1.43	0.03	NS				NS				NS		
*01:01*05:03-*05:01	0	0.0	1	1.43	0.00	NS				NS				NS		
*11:06-*03:01-*05:01	0	0.0	1	1.43	0.00	NS				NS				NS		
Others	0	0.0	0	0.00	24.40	NS				NS				NS		
total	24	100.00	70	100.00	100.00											

Haplotypes with frequencies more than 1.0% in controls were included to the analysis (53 haplotypes). N, n, number of the haplotypes.

*1 Control subjets: HLA LABORATORY, Japan INC: http://hla.or.jp/med/frequency_search/ja/haplo/ ref (11).

*2 Each haplotype frequency was analyzed using Fisher’s exact test with 2 x2 contingency tables.

*3 Pc: Bonferroni correction.

F, frequency of the haplotype; NS, not significant.

P values less than 0.05 are shown in bold.

95%CI: 95% confidence intervals.

In haplotype analysis, HLA-DRB1*04:05-DQB1*04:01 haplotype frequency was significantly higher in patients with ICI-T1DM than those of general controls and also in ICI-controls (P=0.026 and 0.003, respectively) (**Table 2D**). HLA-DRB1*04:05-DQB1*04:01-DPB1*05:01 haplotype frequency was significantly higher in patients with ICI-T1DM than those of general controls and also in ICI-controls (P=0.006 and 0.002, respectively) (**Table 2E**).

Of all significant HLA alleles and haplotypes, notably, only HLA-DQB1*04:01 and HLA-DPB1*05:01 allele frequencies were significantly increased in patients with ICI-T1DM compared with ICI-controls after Bonferroni correction (Pc=0.033 and Pc=0.04, respectively) (**Tables 2B**, **2C**). Then the allele frequencies of HLA-DQB1*04:01 and HLA-DPB1*05:01 were compared in conditional multiple logistic regression analysis (**Table 2F**).

**Table 2F:** Multiple logistic regression analysis of HLA-alleles in ICI-T1DM patients and ICI-controls.

HLA allele	Predicted score	Standard error	P-value	Odds ratio	95%CI
HLA-DQB1*04:01 (completely in linkage disequilibrium with HLA-DRB1*04:05)	0.755	0.333	**0.023**	4.53	1.22--16.7
HLA-DPB1*05:01	0.647	0.268	**0.016**	3.65	1.27--10.44

95%CI: 95% confidence intervals.

P values less than 0.05 are shown in bold.

Both allele frequencies were found to be significantly increased in ICI-T1DM, P=0.023 for HLA-DQB1*04:01 and P=0.016 for HLA-DPB1*05:01 (**Table 2F**). Note that the result also indicates the equivocal importance of following 3 factors: HLA-DQB1*04:01, HLA-DRB1*04:05, and HLA-DRB1*04:05-DQB1*04:01 haplotype, due to the complete linkage disequilibrium between HLA-DRB1*04:05 and HLA-DQB1*04:01 in the current study population (**Table 2F**).

### Amino acid polymorphisms in each HLA-class II molecules

**Figure 1:**
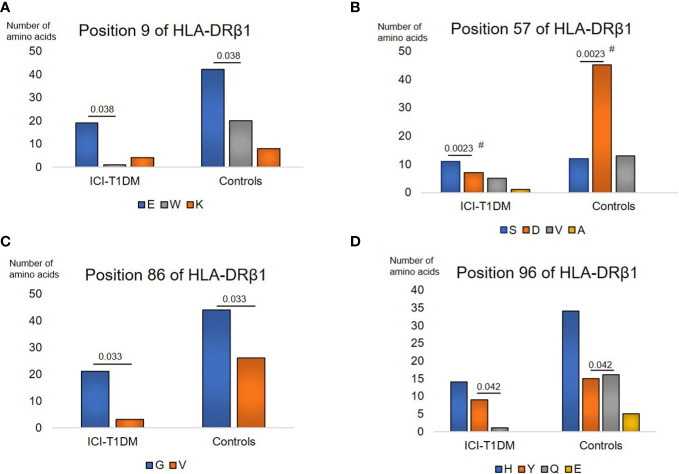
Prevalence of amino acid carriages at residues of HLA-DRB1 allele in patients with ICI-T1DM and ICI-controls (shown as controls) **(A–D)**. Prevalence of amino acid at position 9, E, Glutamic acid vs W, Tryptophan, P=0.038. OR 9.05, 95% CI: 1.13-72.43 **(A)**, position 57, S, Serine vs D, Aspartic acid, P=0.0023. OR 5.89, 95%CI: 1.88-18.46 **(B)**, position 86, G, Glycine vs V, Valine, P=0.033. OR 4.14, 95%CI: 1.12-15.23 **(C)**, and position 96, Y, Tyrosine vs Q, Glutamine, P=0.042. Odds ratio of 9.60, 95%CI: 1.08-85.16 **(D)** are shown. After Bonferroni correction for all significant amino acid polymorphisms among HLA-DRB1 alleles, only amino acid position 57 on HLA-DRB1 allele **(B)** was significantly different (Pc=0.046 after Bonferroni correction, shown with #).

Further, univariate logistic regression analysis was thoroughly performed to examine relationships between ICI-T1DM and amino acid polymorphisms at HLA-DRβ1 (237 amino acid positions) (**Supplementary Table 3A**), DQβ1 (237 amino acid positions) (**Supplementary Table 3B**, and DPβ1 (229 amino acid positions) (**Supplementary Table 3C**). The amino acid polymorphisms with significance are indicated as yellow in the tables and they underwent further evaluation. Of those, amino acid carriages at amino acid positions 9, 57, 86, and 96 of HLA-DRβ1 were significantly different between the patients with ICI-T1DM and ICI-controls (**Supplementary Table 4A, B**). Glu (E) was significantly more frequently observed than Trp (W) (represented as E>W, the same applies hereafter) at position 9 (**Figure 1A**), Ser (S) > Asp (D) at position 57 (**Figure 1B**), Gly (G) > Val (V) at position 86 (**Figure 1C**), and Tyr (Y) > Gln (Q) at position 96 of HLA-DRβ1 in ICI-T1DM (**Figure 1D**).

**Figure 2:**
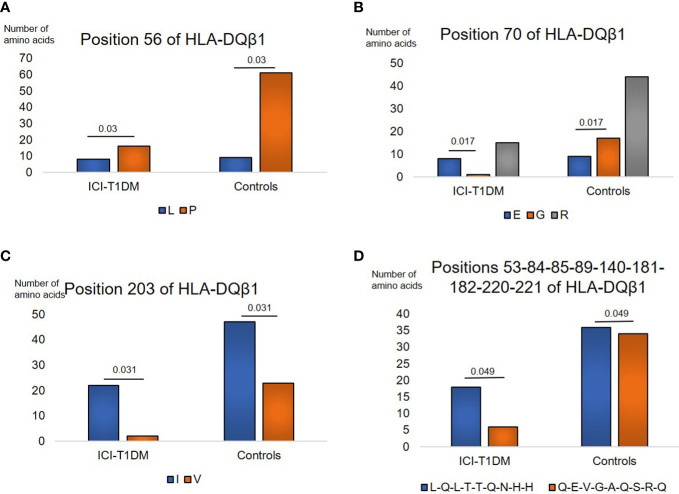
Similarly, prevalence of amino acid carriages at residues of HLA-DQB1 allele in patients with ICI-T1DM and ICI-controls **(A–D)**. Prevalence of amino acid at position 56, L, Leucine vs P, Proline, P=0.03. OR 3.39, 95%CI: 1.13-10.18 **(A)**, position 70, E, Glutamic acid vs G, Glycine, P=0.017. OR 15.11, 95%CI: 1.62-140.58 **(B)**, position 203, I, Isoleucine vs V, Valine, P=0.031. OR 5.38, 95%CI: 1.16-24.89 **(C)**, and positions 53-84-85-89-140-181-182-220-221, L-Q-L-T-T-Q-N-H-H, Leucine-Glutamine-Leucine-Threonine-Threonine-Glutamine-Asparagine-Histidine-Histidine vs Q-E-V-G-A-Q-S-R-Q, Glutamine-Glutamic acid-Valine-Glycine-Alanine-Glutamine-Serine-Arginine-Glutamine, P=0.049. OR 2.83, 95%CI: 1.01-7.98 **(D)** are shown. After Bonferroni correction for all significant amino acid polymorphisms among HLA-DQB1 alleles, no amino acid polymorphisms were significantly different.

Regarding HLA-DQβ1, amino acids carriages at amino acid positions 56, 70, 203, and 53-84-85-89-140-181-182-220-221 (each amino acid in complete linkage disequilibrium) of HLA-DQβ1 were significantly different between the patients with ICI-T1DM and ICI-controls (**Supplementary Tables 5A, B**). Leu (L) > Pro (P) at position 56 (**Figure 2A**), E > G at position 70 (**Figure 2B**), Ile (I) > V at position 203 (**Figure 2C**), and Leu-Gln-Leu-Thr-Thr-Gln-Asn-His-His (L-Q-L-T-T-Q-N-H-H) > Gln-Glu-Val-Gly-Ala-Gln-Ser-Arg-Gln (Q-E-V-G-A-Q-S-R-Q) at positions 53-84-85-89-140-181-182-220-221 of HLA-DQβ1 in ICI-T1DM (**Figure 2D**).

**Figure 3:**
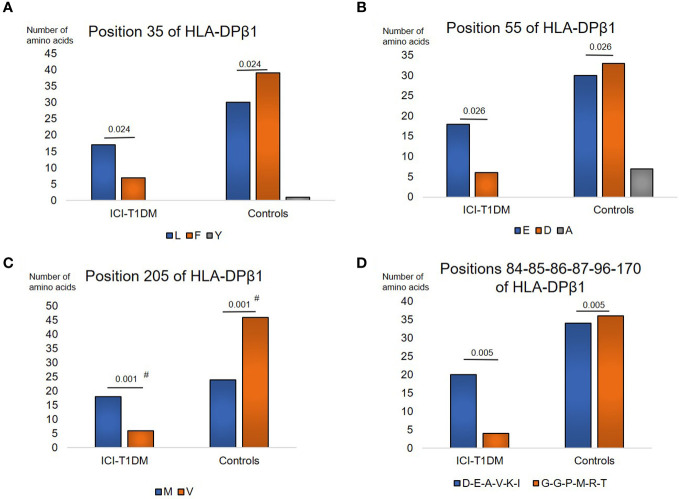
Then, prevalence of amino acid carriages at residues of HLA-DPB1 allele in patients with ICI-T1DM and ICI-controls **(A–D)**. Prevalence of amino acid at position 35, L, Leucine vs F, Phenylalanine, P=0.024. OR 3.16, 95%CI: 1.16-8.59 **(A)**, position 55, E, Glutamic acid vs D, Aspartic acid, P=0.026. OR 3.3, 95%CI: 1.16-9.41 **(B)**, position 205, M, Methionine vs V, Valine, P=0.001. OR 5.75, 95%CI: 2.02-16.39 **(C)**, and positions 84-85-86-87-96-170, D-E-A-V-K-I, Aspartic acid-Glutamic acid-Alanine-Valine-Lysine-Isoleucine vs G-G-P-M-R-T, Glycine-Glycine-Proline-Methionine-Arginine-Tryptophan, P=0.005. OR 5.29, 95%CI: 1.64-17.08 **(D)** are shown. OR, odds ratio; 95%CI, 95% confidence interval; Note that due to the statistical analyses employed, each P value was the same value in the respective figures. After Bonferroni correction for significant amino acid polymorphisms among HLA-DPB1 alleles, only amino acid position 205 on HLA-DP allele **(C)** were significantly different (Pc=0.011 after Bonferroni correction, shown with #).

Moreover, amino acids carriages at amino acid positions 35, 55, 205, and 84-85-86-87-96-170 (each amino acid in complete linkage disequilibrium) of HLA-DPβ1 were significantly different between the patients with ICI-T1DM and ICI-controls (**Supplementary Tables 6A, B**). L > Phe (F) at position 35 (**Figure 3A**), E > D at position 55 (**Figure 3B**), Met (M) > V at position 205 (**Figure 3C**), and Asp-Glu-Ala-Val-Lys-Ile (D-E-A-V-K-I) > Gly-Gly-Pro-Met-Arg-Thr (G-G-P-M-R-T) at positions 84-85-86-87-96-170 of HLA-DPβ1 in ICI-T1DM (**Figure 3D**).

After Bonferroni correction for all significant amino acid polymorphisms, β57 at HLA-DRβ1 (Pc=0.046 by Bonferroni correction, shown with #) (**Figure 1B**), and β205 at HLA-DPβ1 were significantly increased in patients with ICI-T1DM compared with ICI-controls (Pc=0.011 by Bonferroni correction, shown with #) (**Figure 3C**).

To investigate the importance of amino acid polymorphisms mentioned above in detail (**Figures 1**–**3**), a stepwise selection of covariate amino acid residues was applied in multivariate logistic analysis across the HLA-DR, DQ, and DP (**Table 3**). Subsequently, β205 at HLA-DPβ1 was found to be most significant among them.

**Table 3:** Stepwise selection and multivariate regression analysis of amino acid polymorphisms across HLA-class II alleles in ICI-T1DM patients and ICI-controls.

Amino acid position	HLA-chain	Amino acid	Compared amino acid	Predicted score	Test statistics by Wald method	P-value	Selection	Standard error	Odds ratio	95%CI
9	HLA-DRβ1	K and E	W	0	0.044	0.833				
9	HLA-DRβ1	K	E	0	0.070	0.965				
57	HLA-DRβ1	A and S	V and D	0	1.369	0.242				
57	HLA-DRβ1	A	S	0	2.479	0.290				
57	HLA-DRβ1	V	D	0	0.109	0.947				
86	HLA-DRβ1	G	V	0.556	2.499	0.114	Selected	0.352	3.039	0.765--12.061
96	HLA-DRβ1	Y and H	Q and E	0.924	2.919	0.088	Selected	0.541	2.519	0.873--7.269
96	HLA-DRβ1	Y	H	0	1.219	0.270				
96	HLA-DRβ1	Q	E	0	0.485	0.486				
53-84-85-89-140-181-182-220-221	HLA-DQβ1	L-Q-L-T-T-Q-N-H-H	Q-E-V-G-A-Q-S-R-Q	0	0.213	0.644				
56	HLA-DQβ1	L	P	0	0.415	0.519				
70	HLA-DQβ1	E	R and G	0	0.415	0.519				
70	HLA-DQβ1	R	G	0	0.002	0.999				
203	HLA-DQβ1	I	V	0	0.445	0.505				
35	HLA-DPβ1	L	F and Y	0	2.348	0.125				
35	HLA-DPβ1	F	Y	0	0.019	0.991				
55	HLA-DPβ1	E	D and A	0	1.440	0.230				
55	HLA-DPβ1	D	A	0	2.583	0.275				
84-85-86-87-96-170	HLA-DPβ1	D-E-A-V-K-I	G-G-P-M-R-T	0	0.709	0.400				
205	HLA-DPβ1	M	V	0.69	6.001	0.014*	Selected	0.282	3.976	1.318--11.998

K, Lysine; E, Glutamic acid; A, Alanine; S, Serine; V, Valine; G, Glycine; Y, Tyrosine; H, Histidine;

Q, Glutamine; L, Leucine; R, Arginine; I, Isoleucine; F, Phenylalanine; D, Aspartic acid;

M, Methionine; W, Tryptophan; P, Proline; T, Threonine; N, Asparagine; C, Cysteine.

A stepwise selection (cut-off P value at 0.2) of covariate amino acid residues is shown.

*β205 at HLA-DPβ1 was found to be significant (P=0.014, sensitivity 0.75, specificity 0.73, and Area Under the Curve, 0.782)

95%CI: 95% confidence intervals.

**Figure 4:**
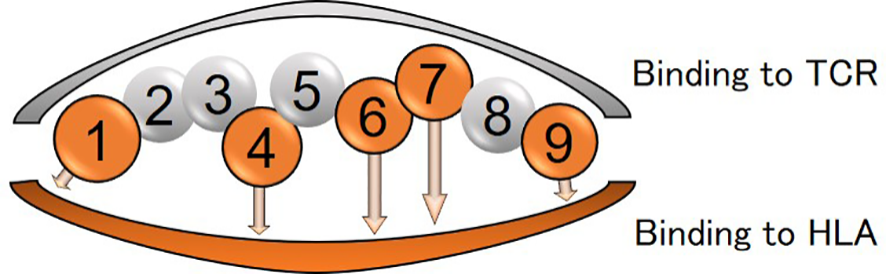
Peptide-binding grooves in an HLA class-II molecule (amino acid position 1-9) are shown. Amino acids in positions 1, 4, 6, 7, and 9 bind to HLA and those in positions 2, 3, 5, and 8 are assumed to be outward facing in order to stimulate the T-cell receptor (TCR).

Differences in amino acids at HLA-DPB1*05:01 and DPB1*15:01 were shown in **Supplementary Table 3C** and **Supplementary Table 6C**). Of those, amino acid at positions 85 and 86 compose pocket 1 (P1) of peptide-binding groove on the HLA molecule, and amino acid at position 9 composes P9 (**Figure 4**).

**Figure 5:**
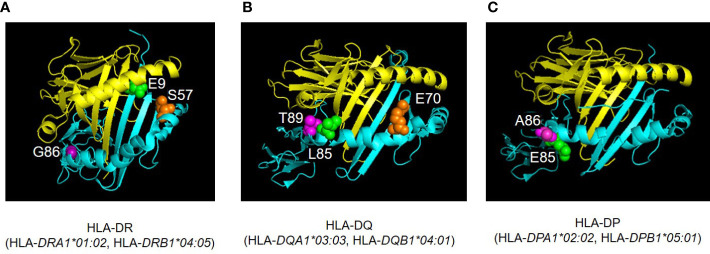
Three-dimensional illustration of ICI-T1DM risk-associated amino acid positions were identified in the current study. The crystal structures of HLA-DR **(A)**, HLA-DQ **(B)**, and HLA-DP **(C)** molecules are established based on Protein Data Bank entries 4IS6, 2NNA, and 3WEX, respectively. The structure of extracellular domains of HLA-class IIα and IIβ chains are shown in yellow and cyan, respectively. Amino acid polymorphic sites are shown as spheres. Amino acid position 9 (E, Glutamic acid, green) and 57 (S, Serine, orange) are located in pocket 9 of HLA-DR molecule (HLA-DRA1*01:02, HLA-DRB1*04:05) **(A)**. Amino acid position 86 (G, Glycine, purple) composes pocket 1 **(A)**. Amino acid position 70 (E, Glutamic acid, orange) is associated with both pocket 4 of HLA-DQ (HLA-DQA1*03:03, HLA-DQB1*04:01) **(B)**. The HLA-DQβ1 amino acid positions 85 (L, Leucine, green) and 89 (T, Threonine, purple) compose pocket 1 of HLA-DQ molecule **(B)**. Amino acid positions 85 (Glu, Glutamic acid, green) and 86 (A, Alanine, purple) compose pocket 1 of HLA-DP molecule (HLA-DPA1*02:02, HLA-DPB1*05:01) **(C)**. Pocket: peptide-binding groove pocket.

Amino acid residues located in the nine peptide-binding grooves (referred to as pockets) were previously described (17, 18). Amino acids at positions 9 and 57 of HLA-DRβ1 compose P9, and the amino acid at position 86 composes P1 as well (**Figures 1A, B, C**, **4**, **5A**). The amino acid at position 70 of HLA-DQβ1 is associated with P4, and amino acids at positions 85 and 89 of HLA-DQβ1 compose P1 as well (**Figures 2B, D**, **4**, **5B**). Amino acids at positions 85 and 86 of HLA-DPβ1 compose P1 (**Figure 3D**, **4**, **5C**).

### Epitope predictions and *in vitro* binding of human proinsulin peptides to HLA-DP5 and HLA-DP15

**Figure 6:**
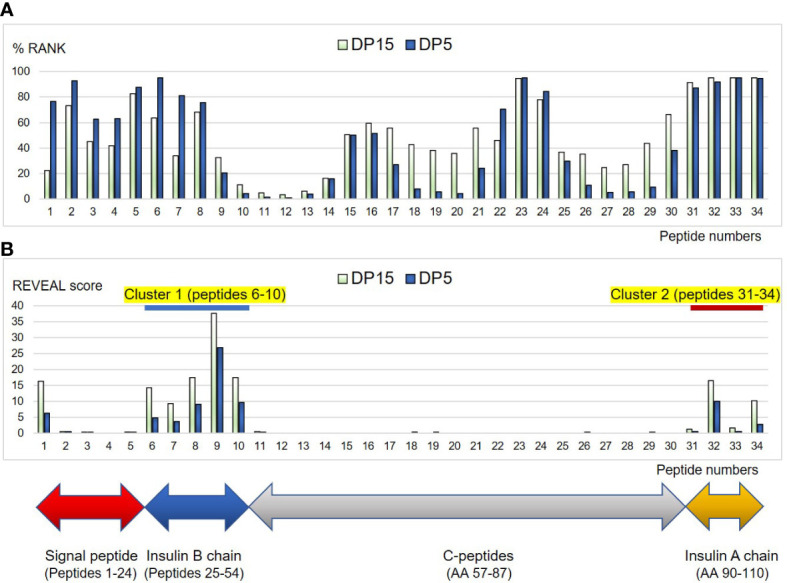
Binding affinities of the peptides derived from human proinsulin to HLA-DP5 (HLA-DPA1*02:02, HLA-DPB1*05:01) or HLA-DP15 (HLA-DPA1*02:02, HLA-DPB1*15:01) molecules are shown as %RANK *in silico* **(A)**, and shown as REVEAL score *in vitro* ***(*B*)***. Horizontal numbers indicate peptide numbers used in the study (shown in **Table 4**). Note that lower %RANK means predicted strong binder *in silico*, and higher REVEAL score indicates peptide with high affinity *in vitro*. Cluster 1 (peptides 6-10) and Cluster 2 (peptide 31-34) were shown with blue and red line, respectively.

Regarding binding of HLA-DP5 and human proinsulin, binding affinity of the signal peptide (AA 1-24) was predicted to be low (possessing high %RANK) (**Table 4**) (**Figure 6A**). In the remaining region (AA 25-110), two epitope candidate regions (AA 43-60 and AA 53-67) were predicted. Five peptides in the regions were strong binders. Other 13 peptides were predicted as intermediate binders. In addition to the 18 peptides, 16 overlapping peptides were synthesized to cover the whole portion. Subsequently, a total of 34 overlapping 15-mer human proinsulin-derived peptides were synthesized and subjected to *in vitro* peptide binding assay (**Table 4**). Besides, proinsulin epitope binding predictions to HLA-DP15 were similarly shown in **Table 4**. The proinsulin binding predictions to HLA-DR5 or to HLA-DP15 were different, but all portions in proinsulin including C-peptide region (AA57-87) showed binding predictions to both alleles.

**Table 4:** Human proinsulin-derived peptides and their affinities to HLA-DP5 and HLA-DP15 *in silico* and *in vitro*.

																	Predicted binding score (shown as %RANK)		In vitro binding score (shown as REVEAL score)		%RANK note	REVEAL score note	References
ID	Position	Amino acid sequences of synthesized peptides															DP15	DP5	DP15	DP5	DP5	DP5	
1	*1-15	**M**	**A**	**L**	**W**	**M**	**R**	**L**	**L**	P	L	L	A	L	L	A	22.17	76.22	16.1	6.1			
2	*7-21	L	L	P	**L**	**L**	**A**	**L**	**L**	**A**	**L**	**W**	**G**	P	D	P	73.14	92.69	0.4	0.4			
3	*13-27	L	L	A	L	**W**	**G**	**P**	**D**	**P**	**A**	**A**	**A**	**F**	V	N	44.91	62.68	0.1	0.2			
4	*19-33	P	D	P	**A**	**A**	**A**	**F**	**V**	**N**	**Q**	**H**	**L**	C	G	S	41.54	63.13	0	0			
5	*25-39	F	V	N	**Q**	**H**	**L**	**C**	**G**	**S**	**H**	**L**	**V**	E	A	L	82.17	87.5	0.3	0.2			
**6**	*29-43	H	L	C	G	S	H	**L**	**V**	**E**	**A**	**L**	**Y**	**L**	**V**	**C**	63.42	95	14.2	4.6		**Cluster 1**	
**7**	*33-47	S	H	**L**	**V**	**E**	**A**	**L**	**Y**	**L**	**V**	**C**	G	E	R	G	33.96	80.96	9.1	3.6			(19)
**8**	*37-51	E	A	L	**Y**	**L**	**V**	**C**	**G**	**E**	**R**	**G**	**F**	F	Y	T	67.91	75.54	17.3	8.9			
**9**	*42-56	V	C	G	E	R	G	**F**	**F**	**Y**	**T**	**P**	**K**	**T**	**R**	**R**	32.28	20.09	37.4	26.8	IB		
**10**	*43-57	C	G	E	R	G	**F**	**F**	**Y**	**T**	**P**	**K**	**T**	**R**	**R**	E	10.81	4.24	17.2	9.5	SB		
11	*44-58	G	E	R	G	**F**	**F**	**Y**	**T**	**P**	**K**	**T**	**R**	**R**	E	A	4.62	1.43	0.4	0.2	SB		
12	*45-59	E	R	G	**F**	**F**	**Y**	**T**	**P**	**K**	**T**	**R**	**R**	E	A	E	3.37	0.71	0	0	SB		
13	*46-60	R	G	**F**	**F**	**Y**	**T**	**P**	**K**	**T**	**R**	**R**	E	A	E	D	5.96	3.74	0	0	SB		
14	*47-61	G	**F**	**F**	**Y**	**T**	**P**	**K**	**T**	**R**	**R**	E	A	E	D	L	16.23	15.52	0	0	IB		
15	*48-62	F	F	**Y**	**T**	**P**	**K**	**T**	**R**	**R**	**E**	**A**	E	D	L	Q	50.27	49.79	0	0	IB		
16	*49-63	F	Y	T	**P**	**K**	**T**	**R**	**R**	**E**	**A**	**E**	**D**	L	Q	V	59.35	51.18	0	0			
17	*50-64	Y	T	P	K	T	R	**R**	**E**	**A**	**E**	**D**	**L**	**Q**	**V**	**G**	55.66	26.85	0	0	IB		
18	*51-65	T	P	K	T	R	**R**	**E**	**A**	**E**	**D**	**L**	**Q**	**V**	**G**	Q	42.39	7.81	0	0.3	IB		
19	*52-66	P	K	T	R	**R**	**E**	**A**	**E**	**D**	**L**	**Q**	**V**	**G**	Q	V	38.06	5.25	0	0.1	IB		
20	*53-67	K	T	R	**R**	**E**	**A**	**E**	**D**	**L**	**Q**	**V**	**G**	Q	V	E	35.72	4.26	0	0	SB		
21	*54-68	T	R	**R**	**E**	**A**	**E**	**D**	**L**	**Q**	**V**	**G**	Q	V	E	L	55.52	23.95	0	0	IB		
22	*59-73	E	D	**L**	**Q**	**V**	**G**	**Q**	**V**	**E**	**L**	**G**	G	G	P	G	45.97	70.3	0	0			
23	*64-78	G	Q	**V**	**E**	**L**	**G**	**G**	**G**	**P**	**G**	**A**	G	S	L	Q	94.37	95	0	0			
24	*69-83	G	G	G	P	G	**A**	**G**	**S**	**L**	**Q**	**P**	**L**	**A**	**L**	E	77.82	84.31	0	0			
25	*74-88	A	G	S	**L**	**Q**	**P**	**L**	**A**	**L**	**E**	**G**	**S**	L	Q	K	36.67	29.36	0	0	IB		
26	*75-89	G	S	L	Q	P	**L**	**A**	**L**	**E**	**G**	**S**	**L**	**Q**	**K**	R	35.2	10.52	0	0.1	IB		
27	*76-90	S	L	Q	P	**L**	**A**	**L**	**E**	**G**	**S**	**L**	**Q**	**K**	R	G	24.48	5.07	0	0	IB		
28	*77-91	L	Q	P	**L**	**A**	**L**	**E**	**G**	**S**	**L**	**Q**	**K**	R	G	I	26.62	5.24	0	0	IB		
29	*78-92	Q	P	**L**	**A**	**L**	**E**	**G**	**S**	**L**	**Q**	**K**	R	G	I	V	43.33	9.38	0	0.1	IB		
30	*79-93	P	**L**	**A**	**L**	**E**	**G**	**S**	**L**	**Q**	**K**	R	G	I	V	E	65.94	37.94	0	0	IB		
**31**	*84-98	G	S	L	Q	**K**	**R**	**G**	**I**	**V**	**E**	**Q**	**C**	**C**	T	S	91.03	86.88	1.1	0.5		Cluster 2	
**32**	*88-102	K	R	G	**I**	**V**	**E**	**Q**	**C**	**C**	**T**	**S**	**I**	C	S	L	94.91	91.7	16.3	9.9			(20)
**33**	*92-106	V	E	Q	C	**C**	**T**	**S**	**I**	**C**	**S**	**L**	**Y**	**Q**	L	E	95	95	1.5	0.5			
**34**	*96-110	C	T	S	**I**	**C**	**S**	**L**	**Y**	**Q**	**L**	**E**	**N**	Y	C	N	95	94.28	10.1	2.6			

Core 9 amino acids in the prediction for HLA-DP5 are shown in bold.

SB, strong binders for HLA-DP5: %RANK<5; IB, intermediate binder for HLA-DP5: %RANK was between 5 to 50.

K, Lysine; E, Glutamic acid; A, Alanine; S, Serine; V, Valine; G, Glycine; Y, Tyrosine; H, Histidine;

Q, Glutamine; L, Leucine; R, Arginine; I, Isoleucine; F, Phenylalanine; D, Aspartic acid; M, Methionine;

W, Tryptophan; P, Proline; T, Threonine; N, Asparagine; C, Cysteine

Peptide numbers within B chain (AA25-54) and A chain (AA90-110) are shown in bold lines.

Then *in vitro* peptide binding assay was conducted, and remarkably, in the insulin B chain (AA25-54) and insulin A chain (AA90-110), two clusters were identified (peptides 6-10: AA 29-57 as cluster 1 and peptides 31-34: AA 84-110 as cluster 2) for HLA-DP5 and also for HLA-DP15 (**Table 4**) (**Figure 6B**). Moreover, peptides in the C-peptide region (AA57-87) bound to neither HLA-DP5 nor HLA-DP15.

## Discussion

A total of 47 patients with malignancies who had been treated with ICI were subjected to HLA typing by next generation sequencing. T1DM developed in twelve and the remaining thirty-five served as controls. In allele and haplotype analyses, the patients had an increase of HLA-DRB1*04:05, DQB1*04:01, and in particular of DPB1*05:01. Indeed, 17/24 (71%) alleles were DPB1*05:01 among the patients compared with 24/70 (34%) in the controls. Moreover, significant amino acid polymorphisms at HLA-DR, DQ, and DP allele were identified that might contribute to the development of ICI-T1DM, probably with conformational alterations in the peptide-binding groove in each HLA-class II molecule. HLA-DP5 was found to be more strongly related to amino acid polymorphisms. As the peptide-binding groove dictate peptide binding, we scanned proinsulin *in silico* as a hypothetical autoantigen and novel human proinsulin epitope clusters in insulin B and A chains were discovered for HLA-DP *in vitro*. Our findings are promising suggestion of a possible association between HLA and ICI-T1DM through proinsulin peptide binding studies.

In comparison with our previous study (5), the current study was conducted on a larger scale with confirmation of HLA-DPB1*05:01 predominance in ICI-T1DM than those of general controls and ICI-controls with the same ethnic background. Current study may also support other reports describing that HLA-DR4 alleles are increased in patients with ICI-T1DM in the United States (21, 22), although these studies are not at the detailed allelic levels. Based on the conditional multiple regression analysis, in addition to HLA-DPB1*05:01 allele, alleles of HLA-DRB1*04:05 and DQB1*04:01, and HLA-DRB1*04:05-DQB1*04:01 haplotype were found to be susceptible to ICI-T1DM (**Tables 2A**–**E**) . HLA-DRB1*04:05-DQB1*04:01 haplotype and HLA-DRB1*04:05-DQB1*04:01-DPB1*05:01 haplotype predominance in ICI-T1DM also suggested that HLA-DP5 controls immune reaction of HLA-*DR* and *DQ* as mentioned below.

Then we disentangled amino acid polymorphisms at positions 9 and 57 (P9) and 86 (P1) of HLA-DRβ1, position 70 (P4 and P7) and positions 85 and 89 (P1) of HLA-DQβ1, and positions 85 and 86 (P1) of HLA-DPβ1 (**Figures 5A–C**). These amino acids contribute to the formation of the peptide-binding grooves on HLA-class II molecules, therefore may functionally contribute to the epitope presentations. Notably, patients with ICI-T1DM in Japan have been reported to have HLA-DRB1*11:01/*13:02 (23), DRB1*04:05 (24–26), DRB1*04:06 (26), and DRB1*09:01 (25), and all of patients above had G86 at HLA-DRβ1. Particularly, both HLA-DRB1*04:05 and DRB1*09:01 have G86 at HLA-DRβ1, and were reported to be associated with T1DM (27). Conversely, HLA-DRB1*15:01, a protective allele for T1DM has V86 at HLA-DRβ1 (27), and HLA-DRB1*15:01 was not seen in patients with ICI-T1DM in the current study. Therefore, G86 and V86 seemed to be disease-promotion and protection alleles, respectively, at HLA-DRβ1, in association with (P1). Todd, et al. reported strongly conserved Asp (D) at position 57 (P1, P9) of HLA-DQβ1 with disease susceptibility in patients with T1DM (28), and D at position 57 (P1, P9) of HLA-DRβ1 seemed to play a strong protective role in ICI-T1DM in the current study (**Figure 5A**). Then, stepwise selection and multivariate analysis revealed that β205 at HLA-DPβ1 was most important (**Table 3**). Therefore, we speculated the amino acid polymorphism β205 at HLA-DPβ, which locates outside of the peptide-binding groove in HLA-DPβ1, may be related to interactions such as HLA-DM, or to alterations in signal transduction within the HLA molecule.

Taken together, significances of HLA-DP5 in ICI-T1DM were observed. Next, we conducted human proinsulin peptide binding prediction to HLA-DP molecules to ensure effective epitope presentation in ICI-T1DM (**Table 4**) (**Figure 6A**). Predicted binding affinities and *in vitro* binding results were quite different for HLA-DP5, especially in the C-peptides region, for reasons unknown (**Figures 6A, B**). Remarkably, insulin B chain epitope (cluster 1): AA 29-57, and the insulin A chain epitope (cluster 2): AA 84-110 were established *in vitro* binding assay (**Figure 6B**). Among them, peptide 9 (AA 42-56), VCGERGFFYTPKTRR (core sequence underlined), was predicted as intermediate binders and also exhibited the strongest *in vitro* peptide binding, thus is mostly expected to be immunogenic T-cell epitope. Insulin peptide B9-23 (AA 33-47) has been reported to be a major autoantigen to induce immunity in the nonobese diabetic mouse, and is also included in the cluster 1 (19). Mannering et al. reported that cells transfected with HLA-DRB1*04:05 presented insulin A1–13 (AA 88-102) which is the identical to the peptide 32 (20). HLA-DP15 has not been reported as a risk allele in ICI-T1DM, but showed similar *in vitro* proinsulin epitopes to HLA-DP5 (cluster 1 and cluster 2) (**Figure 6B**). Despite the dissimilarities in amino acid sequence between HLA-DPB1*05:01 and HLA-DPB1*15:01, the importance of epitope cluster 1 and cluster 2 was reinforced across the alleles *in silico* and *in vitro* (**Supplementary Table 6C**).

In the development of ICI-T1DM, we hypothesized that the inhibition of immune-checkpoint molecules by ICI induced immunity to pancreatic β-cells, as observed in thyroid follicular epithelial cells during thyroid irAE (5, 29). Antibody-dependent cellular cytotoxicity by ICIs, by cytotoxic T-cells (30), or by both of them, would contribute to the development of ICI-T1DM. We speculate that ICI-T1DM-predisposing HLA may also be involved with malignant diseases. Proinsulin epitope, as well as tumor-associated antigen/neoantigen, could be bound to peptide-binding cleft of HLA, and cross-presented on the surface of antigen-presenting cells due to molecular mimicry (3, 4, 9). Another important topic is the correlation of ICI treatment effectivity and ICI-T1DM. Considering that 8/12 (75%) were ICI-responders in ICI-T1DM group and that generally only 20-30% are responders, common mechanisms between ICI treatment and ICI-T1DM were suggested. Therefore, HLA seemed to be associated with both ICI treatment outcome and risk for an endocrine adverse event. Considering that proinsulin is processing into insulin A chain and B chain, and that epitope clusters were identified in the two chains, evaluation of IAA in the time course may be of interest. As well, anti-GAD65 antibody titers in the course seem to be important.

This study has limitations. Firstly, we have tested proinsulin binding to HLA-DP. Binding studies for HLA-DR and HLA-DQ molecules are also desirable. Secondly, more evidence of immunogenicity of HLA-DP5 from *in vitro* and *in vivo* experiments in comparison with HLA-DR/DQ are preferable to confirm the peptide binding data in this study. Thirdly, better investigation of the frequency of HLA-DPB1*05:01, irrespective of ICI treatment is still desirable. Finally, the study consists of a small sample size, so more data with increased numbers may help to establish our results.

In conclusion, HLA-DP5 as a predisposition molecule, and significant amino acid polymorphisms at HLA-class II molecules in patients with ICI-T1DM were established in this study. Based on the *silico* and *in vitro* proinsulin peptide-binding study, conformational changes in the peptide-binding groove of the HLA-DP molecules may influence the immunogenicity of proinsulin epitopes in ICI-T1DM. These genetic factors may be utilized for prediction of ICI-T1DM, and also could contribute to elucidation of the mechanism of cancer immunotherapy and ICI-T1DM. Therefore, current study offers safer and more effective management and monitoring options for cancer immunotherapy. Further investigations are warranted to elucidate the relation of ICI- treatment effectiveness and development of ICI-T1DM.

## Data availability statement

The datasets presented in this study can be found in online repositories. The names of the repository/repositories and accession number(s) can be found in the article/**Supplementary Material**.

## Ethics statement

The studies involving human participants were reviewed and approved by Red Cross Society Wakayama Medical Center (JRCW), Wakayama Medical University Hospital (WMU), and Nagoya University (NU). The patients/participants provided their written informed consent to participate in this study. Written informed consent was obtained from the individual(s) for the publication of any potentially identifiable images or data included in this article.

## Author contributions

HI: Conceptualization, designed the study. original draft preparation. SM: Visualization, writing- reviewing and editing. All authors performed the clinical studies statistically analyzed the data and wrote the manuscript. All authors approved the final version of the manuscript for publication. All authors contributed to the article and approved the submitted version.
